# Access to childbirth care by adolescents and young people in the Northeastern region of Brazil

**DOI:** 10.1590/S1518-8787.2016050005396

**Published:** 2016-05-06

**Authors:** Érida Zoé Lustosa Furtado, Keila Rejane Oliveira Gomes, Silvana Granado Nogueira da Gama

**Affiliations:** I Hospital Universitário da Universidade Federal do Piauí. Teresina, PI, Brasil; II Programa de Pós-Graduação em Ciências e Saúde. Centro de Ciências da Saúde. Universidade Federal do Piauí. Teresina, PI, Brasil; III Programa de Pós-Graduação em Epidemiologia em Saúde Pública. Escola Nacional de Saúde Pública. Fundação Oswaldo Cruz. Rio de Janeiro, RJ, Brasil

**Keywords:** Pregnancy in Adolescence, Midwifery, Birthing Centers, Health Services Accessibility, Health Inequalities

## Abstract

**OBJECTIVE:**

To identify the factors that interfere with the access of adolescents and young people to childbirth care for in the Northeast region of Brazil.

**METHODS:**

Cross-sectional study with 3,014 adolescents and young people admitted to the selected maternity wards to give birth in the Northeast region of Brazil. The sample design was probabilistic, in two stages: the first corresponded to the health establishments and the second to women who had recently given birth and their babies. The data was collected by means of interviews and consulting the hospital records, from pre-tested electronic form. Descriptive statistics were used for the univariate analysis, Pearson’s Chi-square test for the bivariate analysis and multiple logistic regressions for the multivariate analysis. Sociodemographic variables, obstetrical history, and birth care were analyzed.

**RESULTS:**

Half of the adolescents and young people interviewed had not been given guidance on the location that they should go to when in labor, and among those who had, 23.5% did not give birth in the indicated health service. Furthermore, one third (33.3%) had to travel in search of assisted birth, and the majority (66.7%) of the postpartum women came to maternity by their own means. In the bivariate analysis, the variables marital status, paid work, health insurance, number of previous pregnancies, parity, city location, and type of health establishment showed a significant association (p < 0.20) with inadequate access to childbirth care. The multivariate analysis showed that married adolescents and young people (p < 0.015), with no health insurance (p < 0.002) and from the countryside (p < 0.001) were more likely to have inadequate access to childbirth care.

**CONCLUSIONS:**

Adolescents and young women, married, without health insurance, and from the countryside are more likely to have inadequate access to birth care. The articulation between outpatient care and birth care can improve this access and, consequently, minimize the maternal and fetal risks that arise from a lack of systematic hospitalization planning.

## RESUMO

**OBJETIVO:**

Identificar os fatores que interferem no acesso de adolescentes e jovens à assistência ao parto na região Nordeste do Brasil.

**MÉTODOS:**

Estudo seccional realizado com 3.014 adolescentes e jovens admitidas nas maternidades selecionadas por ocasião da realização do parto na região Nordeste do Brasil. O desenho da amostra foi probabilístico, em dois estágios: o primeiro correspondeu aos estabelecimentos de saúde e o segundo às puérperas e seus conceptos. A coleta de dados foi realizada por meio de entrevista e consulta ao prontuário hospitalar, a partir de formulário eletrônico pré-testado. Utilizou-se estatística descritiva para análise univariada, teste Qui-quadrado de Pearson para análise bivariada, e regressão logística múltipla para análise multivariada. Foram analisadas variáveis sociodemográficas, antecedentes obstétricos e aquelas relacionadas à assistência ao parto.

**RESULTADOS:**

Metade das adolescentes e jovens entrevistadas não foi orientada sobre o local que deveria procurar para o parto, e entre aquelas que foram orientadas, 23,5% não realizaram o parto no serviço de saúde indicado. Além disso, 1/3 (33,3%) teve que peregrinar em busca de assistência ao parto, sendo que a maioria (66,7%) das puérperas chegou à maternidade por meios próprios. Na análise bivariada, as variáveis situação conjugal, trabalho remunerado, plano de saúde, número de gestações anteriores, paridade, localização do município e tipo de estabelecimento mostraram associação significativa (p < 0,20) com o acesso inadequado à assistência ao parto. Na análise multivariada, adolescentes e jovens que mantinham laços conjugais (p < 0,015), não possuíam plano de saúde (p < 0,002) e eram procedentes do interior (p < 0,001) tinham mais chance de acesso inadequado ao parto.

**CONCLUSÕES:**

Adolescentes e jovens que mantêm laços conjugais, não possuem plano de saúde ou são procedentes do interior têm maior chance de apresentar acesso inadequado ao parto. A articulação entre o atendimento ambulatorial e a assistência ao parto pode melhorar esse acesso e, consequentemente, minimizar os riscos materno-fetais decorrentes da falta de planejamento sistêmico para a internação.

## INTRODUCTION

To meet their requirements in the area of health, users must overcome one essential step: access to these services^18^. Studies on access to health services can make a contribution regarding the organization of care, which enable levels of coverage and identification of priority populations to be established in health care. In addition, these studies are useful in terms of establishing public policies that ensure universal and equal access to health care and in the reorganization of their systems, all of which can minimize inequalities in health^10^.

During the 1990s, Brazil saw an expansion in the primary health care system, marked by the implementation of the Community Health Agents Program and the Family Health Program. Following the year of 2000, the income gap between the rich and poor began to decrease, which can mostly be attributed to income transfer programs and the increase in wages for unskilled labor. In addition, large investments were made in basic education during the 1990s, which resulted in an improvement in the education levels of Brazilian mothers that had never been previously reached. These changes had a role in improving the level of care provided during labor and childbirth in Brazil^22^.

Despite the level of prenatal and birth care being high, these care stages remain poorly integrated. It is difficult for pregnant women to be hospitalized during their child’s delivery, a fact that is still relevant to maternal and child morbidity and mortality and reflects a lack of organization of the health system as an effective referral, counter-referral, transportation and communication system. This problem is a common occurrence in public institutions, where it is difficult to establish a link between pregnant women and the health system. The result of this separation is that the history of the pregnancy is not known and that there is insufficient planning regarding hospitalizations during childbirth^4^ ^,^ ^13^ ^,^ ^15^.

In Latin America and the Caribbean, the largest proportion of childbirths that were given medical and hospital care were seen in the Dominican Republic, Brazil and Colombia^19^. Considering that 95.0% of births in Brazil take place in hospitals, discussing access to maternity wards is a pertinent subject. The greater the distance that the pregnant woman has to travel, the harder it is for her to access health services and, during this potentially lengthy trip, the ongoing labor becomes complicated and risky. This situation can reduce the chances of the pregnant women receiving suitable care, since many health services are not equipped to deal with the more complex cases – which is especially true in the Northeast region of Brazil, where the number of neonatal intensive care units per 1,000 live births is the smallest in the country^2^ ^,^ ^4^.

Thus, determining the risk of a pregnancy becomes useless if the woman does not have access to the maternity ward in a timely manner. Some of the reasons for this delay include the long distances to be travelled and the lack of an effective transport system, as well as poor communication and referral systems^15^.

The objective of our study is to identify factors that interfere with access to childbirth care by adolescents and young women in Brazil’s Northeast region.

## METHODS

This study is cross-sectional in nature, complementing a broader research project performed by the Oswaldo Cruz Foundation (Fiocruz), titled “Born in Brazil: national survey on childbirth”. The methodology used in this study is detailed in Leal et al.^14^ (2012).

The subjects involved in our research were 12 to 24 year-old adolescent and young women who had recently given birth. These women had been admitted to the selected maternity wards in the Northeast region of Brazil to give birth. This age group was chosen because pregnancy in these groups is associated with greater social vulnerability, which is especially true during adolescence. In addition, becoming pregnant during adolescence may result in negative consequences for the mother’s health^20^ and adverse neonatal outcomes such as low birth weight and prematurity, which are considered significant causes of infant and neonatal death^23^. Furthermore, this section of the population experiences difficulties in terms of access to care, such as geographical and economic barriers, in addition to services being offered at times that are incompatible with the users’ needs and an insufficient number of professionals who are ready to care for them^6^.

The study sample was stratified by major geographical regions in Brazil (North, Northeast, Southeast, South, and Midwest), by municipal location (state capital and countryside), and by type of establishment (public, private and mixed), considering that there are variations regarding cesarean sections. The health care establishments were classified according to information contained in the Brazilian National Register of Health Establishments, with the private establishments that have a funding agreement with the Brazilian Unified Health System (SUS) being categorized as mixed.

Thus, 30 sampling strata were created including all the Brazilian states. The selected health establishments in each of the five macro-regions were made up of five substrates: state capital, countryside, public, private, and both public and private (mixed). A two-stage probability sampling method was used in each stratum to define, firstly, the sample corresponding to the health establishments and, subsequently, the new mothers and their conceptions (living newborn or stillborn).

The sample size in each stratum was calculated based on the cesarean section rate in Brazil from 2007, which obtained a minimum sample of 341 women per stratum. As the sampling was done by conglomerates, a design effect of 1.3 was used, totaling a sample of approximately 450 new mothers per stratum. Regarding the health establishments, a total of 266 hospitals were sampled for the study base, with the number of randomly selected establishments ranging from five to 39, depending on the size of the substrate. Within each substratum, the establishments had a probability of being selected that was proportionate to their size. Therefore, each hospital included in the study had the same sample size, which was 90 pairs of new mothers and their babies^14^.

To ensure that the estimates obtained were similar to the number of births from the 2011 population sample, a calibration procedure was performed. This procedure involved weighting the data being calculated by inversing of the inclusion probability of each new mother in the sample. Thus, the results obtained are estimates of the population of new mothers studied (337,451), which are based on the sample of 3,014 adolescents and young people who were interviewed.

The data were collected by previously trained interviewers through the central coordination of the study base between February 2011 and October 2012.

Only health institutions that registered 500 births/year or more were eligible for the study sample. New mothers who conceived infants (alive or dead) aged ≥ 22 gestational weeks or with a birth weight of ≥ 500 g at these establishments participated in this study. Women who delivered their infants at these health facilities but who had severe mental illness, were homeless, foreigners who could not understand or speak Portuguese, individuals with hearing or speech impairment, and those who were prevented from involvement by court order were excluded from the study.

The data were collected from the study base using three basic electronic forms: the first was applied to women who had recently given birth (at least six hours after childbirth); the second was filled based on the clinical chart data, and the third was applied over the phone to the mother between 45 and 60 days after childbirth. For this study, issues of interest were selected and included in the first and second forms.

The selected variables were related to demographic data (age, marital status, education, work status, economic class, and health insurance), obstetric history (number of previous pregnancies and parity) and variables related to childbirth care (information regarding the delivery location, child delivery at the indicated location, care sought elsewhere, method of travel to the maternity ward, and presence of an accompanying person).

The data were analyzed using the Statistical Package for the Social Science (SPSS) software, version 17.0. Descriptive statistics were used for the univariate analysis. For the bivariate analysis, Pearson’s Chi-square test (χ^2^) or Fisher’s exact test, when appropriate, were used. Multiple logistic regression was used to explain the joint effect of the independent variables on the dependent variable – access to childbirth care. The inclusion criterion for variables in the logistic model was an association with socioeconomic characteristics and reproductive aspects with a p-value of < 0.20 in the bivariate analysis^11^. An association with a p-value of < 0.05 in the model was considered.

The term “access” was used to describe the act of including childbirth care within health services in a timely manner, since the difficulty in gaining access harms reaching suitable care and may cause a delay in such, which contributes to additional health problems for the woman and the fetus. Thus, it was considered that the young and adolescent new mothers who traveled in search of childbirth care had inadequate access, while the others did obtain adequate access to health services.

A variance inflation factor of above four was adopted as a cut-off point for diagnosing multicollinearity^a^. However, the test did not detect multicollinearity among the independent variables under study.

Performing this study as a complementary design of the base study was authorized by the central coordination, which was done through design analysis and disclaimer information to use part of the database. The study was submitted and approved by the Fiocruz Ethics and Research Committee (Opinion 92/10) and health institutions from which the data was collected, all in accordance with the ethical-legal guidelines for research involving human beings, as described in Resolution 196/96 of the Brazilian National Health Council.

## RESULTS

The participants involved in this study were aged between 12 and 24 years, with an average age of around 20 years (SD = 2.84), most of whom occupied the 20 to 24 years age group (56.2%). The majority of adolescents and young women mentioned being married (77.6%), having an education level of up to middle school (54.6%) and belonging to economic classes D and E (50.7%). Less than 1/5 of those interviewed had a paying job (16.7%) and almost none had health insurance (90.9%). A significant portion of the adolescents and young women were from the countryside (79.7%) and more than half received care through SUS (53.1%). Regarding reproductive aspects, 58.1% were first-time mothers and, among those who had become pregnant before, 22.8% had underwent only one other birth (Table 1).

**Table 1:** Sociodemographic characterization and reproductive history of the new adolescent and young mothers in the Northeast region of Brazil, 2012.

Variable	n	%
Sociodemographic characterization		

Age group (years)		
12-19	1,320	43.8
20-24	1,694	56.2
Marital status		
No martial ties	674	22.4
With martial ties	2,337	77.6
Education		
Complete elementary school or less	1,642	54.6
Incomplete high school and over	1,363	45.4
Working		
No	2,511	83.3
Yes	502	16.7
Economic class		
A+B+C	1,474	49.3
D+E	1,516	50.7
Health insurance		
No	2,735	90.9
Yes	274	9.1
Origin		
Countryside	2,401	79.7
Capital city	613	20.3
Type of establishment		
Brazilian Unified Health System	1,599	53.1
Mixed	1,276	42.3
Private	139	4.6

Reproductive history		

Number of previous pregnancies		
First pregnancy	1,749	58.1
One	748	24.8
Two or more	515	17.1
Parity		
Primipara	1,956	64.9
One previous childbirth	687	22.8
Two or more previous childbirths	371	12.3

Inadequate access to childbirth care was a reality for 32.2% of the new mothers (adolescents and young women). As for the characterization of the population about access to childbirth care, 50.0% of the interviewed women were not informed as per where they should go to give birth. Among those who were informed, 23.5% did not give birth at the health service that was indicated and one third (33.3%) had to look for alternative childbirth care. Two in three of the new mothers (66.7%) traveled to the maternity ward by their own or other means, and 76.7% reported being accompanied by another individual during hospitalization (Table 2).

**Table 2:** Distribution of new mothers according to variables related to access and delivery location. Northeastern region of Brazil, 2012.

Variable	%	CI95%
Inadequate access to childbirth care	32.2	
Delivered at the indicated location*		
Yes	76.5	71.3–81.0
No	23.5	19.0–28.7
Given information regarding the delivery location		
Yes	50.0	46.6–53.9
No	50.0	46.6–53.9
Sought care elsewhere		
Yes	33.3	28.1–38.9
No	66.7	61.1–71.9
Method of traveling to maternity ward		
Own or with others	67.6	63.0–71.8
Ambulance or official vehicles	32.4	28.2–37.0
Accompanying person		
Yes	76.7	70.0–82.3
No	23.3	17.7–30.0

* Question only posed to the women who claimed to have received information regarding the delivery location.

The data in Table 3 shows that inadequate access to childbirth care had a significant association (p < 0.05) with being married, having no health insurance, and being from the countryside.

**Table 3:** Bivariate analysis and multiple logistic regression model for inadequate access to childbirth care by adolescent and young new mothers. Northeastern region of Brazil, 2012.

Variable	Inadequate access to childbirth care				

	Bivariate			Multivariate	
	
	Yes (%)	No (%)	p^a^	ORa (95%CI)	p^b^
Age group (years)					
12-19	34.6	65.4	0.362	-	-
20-24	32.3	67.7			
Marital status					
Single	28.5	71.5	**0.016**	1	**0.015**
Married	34.7	65.3		1.34 (1.05–1.71)	
Reported skin color					
White	33.2	66.8	0.975	-	-
Non-White	33.3	66.7		-	
Literate					
No	36.1	63.9	0.673	-	-
Yes	33.3	66.7		-	
Education					
Complete elementary school or less	33.2	66.8	0.952	-	-
Incomplete high school and over	33.4	66.6		-	
Working					
No	34.1	65.9	**0.062**		
Yes	29.2	70.8			
Economic class					
A+B+C	31.8	68.2	0.241	-	-
D+E	35.1	64.9		-	
Health insurance					
No	34.7	65.3	**< 0.001**	1	**0.002**
Yes	20	80		0.45 (0.28–0.73)	
Origin					
Capital city	31.5	68.5	**< 0.001**	1	**< 0.001**
Countryside	40.5	59.6		1.26 (1.42–3.58)	
Type of establishment					
SUS	26.9	73.1			
Mixed	44.1	55.9	**< 0.001**	-	-
Private	9.4	90.6		-	
No. previous pregnancies					
Up to one	34.4	65.6	**0.060**		
Two or more	28	72			
Parity					
Up to one	34	66	**0.179**	-	-
Two or more	28.2	71.8		-	
Previous abortion					
No	30.1	69.9	0.542	-	-
Yes	33.2	66.8		-	
Number of previous miscarriages					
Up to one	30.7	69.3	0.519	-	-
Two or more	37.7	62.3		-	
Planned pregnancy					
No	31.2	68.8	0.280	-	-
Yes	33.2	66.8		-	

^a^ Chi-square or Fisher Test, p < 0.200 significance level.

^b^ Multiple Logistic Regression, p < 0.05 significance level.

Statistically significant values are in bold font.

## DISCUSSION

Adolescents and young women who were married, who did not have health insurance, or who were from the countryside had a higher chance of experiencing inadequate access to childbirth health care. In addition, half of the new mothers were not informed about the place to give birth, and among those who were, a significant portion were not able to get care at the indicated location. Pregnancy during adolescence is more common among lower-income groups with a low level of education and family income. This group becomes sexually active, gets pregnant, and gets married earlier in life when compared to other groups of women^12^ ^,^ ^16^. The Northeastern region of Brazil is considered one of the poorest areas of the Country, and its inhabitants find it most difficult to access health services^16^, which may explain the high incidence of adolescent pregnancy, as well as the precarious socioeconomic conditions of the participants from this research.

Demand for health services results from the population’s combined social, cultural, and individual situations. The determinants for using these types of services include the sociodemographic characteristics of the individuals in question^3^. In Brazil, groups that have the greatest health needs are precisely those that have the most difficulty accessing and using health care services^21^.

This study found that married adolescents and young women who had recently given birth have a higher propensity for inadequate access to childbirth care. What may have contributed to this association is that certain social conditions, such as marriage and family commitment, coupled with a low socioeconomic level and lack of paid work, make it impossible for these women to pay someone to take care of their house and existing children, which can have a negative influence on how these women look after themselves.

We observed that not having health insurance has a significant association with inadequate access to childbirth care. Having health insurance has been demonstrated to be directly related to family income^5^. Public health services have also been shown to be mostly used by individuals with poor socioeconomic conditions, with SUS caring for more than 80.0% of adolescent pregnant women^7^ ^,^ ^16^.

One study that investigated access to health services by adolescents and adults in the city of Pelotas, Rio Grande do Sul, Brazil, noted that the demand for health care services seemed low, in particular from the section of the population with no health insurance, and that concentration of this demand came from poorer groups. This evidence suggests that the inequality in access to health services in Brazil is a result of the institutional arrangement that allows more access to the system by individuals who are able to pay for it. Similarly, empirical international evidence supports the idea of there being inequalities in societies that opt for mixed financing in health systems^17^.

In the Brazilian countryside, adolescents and young women are more likely to have inadequate access to childbirth care. In Brazil, health services are concentrated in urban areas, capitals, and central areas, with the rural, poorer, and peripheral areas being neglected as a result. Thus, the smaller the size of the population, the greater distance the parturient woman has to travel, which is probably due to the concentration of establishments with obstetric beds being in large cities. In addition, socioeconomic and cultural factors play a role in the great inequality in the supply and the increased difficulty of accessing health services^2^.

Therefore, the results from this study reinforce the assumption that the possibility of using a certain type of care is associated with, among other factors, the income of the individual and coverage by health insurance. This scenario reproduces the social inequalities within the system to the extent that the chances of choosing the services are limited by these factors^3^.

However, despite there having been various policies aimed at maternal and child health implemented in recent decades, representing important advances in Brazil’s health indicators, there are still inequalities to be overcome. One example of these inequalities is the inadequate access to childbirth care being significantly associated with not using the health insurance observed in this study. Thus, it is a matter of urgency that all citizens are guaranteed access to good quality resolutive public health actions, especially when considering the most economically and socially vulnerable strata of women from the poorest regions of Brazil, most of who are SUS users.

Only half of the women in the study were given guidance regarding which hospital unit to head to when going into labor. This percentage was lower than that observed in the 2009 national survey, which showed that around 60.0% of mothers reported having been given this advice, while almost 90.0% of births took place at the first maternity unit that the mothers found during labor^16^. Our result reinforces what was observed during a research project performed in the municipality of Rio de Janeiro^8^ and denounces the fragility or even absence of a regionalized care system focused on childbirth. The result also highlights the inadequate supply of beds in light of demand, and may increase maternal and neonatal risks due to possible obstetric complications that contribute to timely care, which could prevent fatal complications, not being available.

The Northeast region of Brazil has the lowest percentage of all regions regarding to this guidance, despite it being the responsibility of the health professionals who care for these women to provide it^16^. Nevertheless, developing a system that is focused on the child-bearing process and childbirth itself to help organize this flow depends on what the competent authorities can organize. However, doing so only makes sense in cities or micro-regions in which there is more than one maternity unit, because the lack of such organization transfers the burden of searching for and finding maternity hospitals with available beds, when going into labor, for women^16^.

Even among those who received guidance, there are a significant number of adolescents and young women who are not able to reach the indicated health service, with about one third of these individuals going to another service to seek care. In this way, continuous care during the prenatal and childbirth periods, despite their importance and legal support, through Law 11,634, of December 27, 2007^b^, has not been achieved. Even when the pregnant women look for the hospitals that they have been referred to, the binomial prenatal-postpartum is not always covered by these services, since the basic units perform formal referrals to other hospitals, which do not always happen^1^.

The situation is even more critical when patients have to travel around looking for care at other establishments by their own means^4^ ^,^ ^15^. In this study, less than one third of the interviewees were transported to the care unit by ambulance, with the other two thirds having to find a suitable place to give birth on their own, which reinforces the lack of hierarchy in the network, despite there being an adequate communication and transport system in the Northeast region of Brazil. In addition to the weakness of health care during pregnancy being shown by the little expectation of hospitalization, there can still be harm for parturient women, who are often in labor or in risky situations, while the long distances traveled to find a health unit to give birth constitute potential risk for the maternal-fetal outcome^4^ ^,^ ^15^.

Another relevant point to consider in childbirth care for adolescents and young women is emotional support to women during labor and birth itself, which is increasingly emphasized when breaking the solitude imposed on her by traditionally established institutional routines, by providing more favorable obstetric and neonatal results^9^. These were the reasons that led to the enactment of law 11,108, April 2005^c^, which ensures that women are not alone during childbirth or postpartum in SUS maternity wards.

The rate at which women have had company has increased substantially when compared to what was observed in 2009, when the Northeast region obtained a lower frequency of this variable in relation to percentages from the rest of Brazil. However, the frequency of this variable is still less than desirable. It is believed that the infrastructural limitation of most Brazilian maternity wards using an accompanying person chosen by the women, without compromising the privacy of others, has hampered the implementation of this measure. There is also resistance from the institutions, which is based on a fear that the presence of an accompanying person will disrupt the established work routine^16^.

Therefore, the health system is constantly encouraged not to consider childbirth care as an isolated procedure that has no link to prenatal care, and to create articulation strategies at the different levels of care complexity. The difficulty of accessing health services leads to preventable deaths that occur in the North and Northeast regions of Brazil, these are both maternal as well as fetal and neonatal, which is in spite of the rate of births occurring in health units in Brazil having increased from 91.0% to 98.0%^16^.

Thereby, the inseparable nature of prenatal and childbirth care is vitally important and must be taken into account by health managers, given the high vulnerability of this population group. In this sense, especially in the poorest regions of Brazil, the implementation of the Brazilian *Rede Cegonha* [Stork Network] government program is expected to expand and qualify the care given to pregnant women and new mothers, thereby providing dignity, humanity, and security during the child-bearing period and birth itself^d^.

Based on the aforementioned, it can be inferred that important reforms must be made regarding childbirth care in the Northeast region of Brazil. Thus, all pregnant women, when going through prenatal care, should be given information about the maternity unit they should go to when in labor. Whenever there are no free beds in the indicated maternity ward, the pregnant women should be forwarded to another central line of maternity beds and must never go looking for another unit by themselves, as is still observed at a significant percentage in the new mothers from our study.

Therefore, this study’s hypothesis was proved correct, there is a challenge for the health system to improve articulation between outpatient care and childbirth care, which is especially true in public institutions, where it is difficult to establish a link between pregnant women and the health system, which in turn leads to a lack of hospitalization planning regarding childbirth.

## Footnotes

a Garson GD. Statnotes: topics in multivariate analysis. Reliegh: NC State University; [s.d.] [cited 11 Jul 2013]. Available from: http://faculty.chass.ncsu.edu/garson/pa765/statnote.htm

b Brasil. Lei nº 11.634, de 27 de dezembro de 2007. Dispõe sobre o direito da gestante ao conhecimento a vinculação à maternidade onde receberá assistência no âmbito do Sistema Único de Saúde. *Diario Oficial Uniao*. 28 dez 2007

c Brasil. Lei nº 11.108, de 7 de abril de 2005. Altera a Lei no 8.080, de 19 de setembro de 1990, para garantir às parturientes o direito à presença de acompanhante durante o trabalho de parto, parto e pós-parto imediato, no âmbito do Sistema Único de Saúde - SUS. *Diario Oficial Uniao*. 8 abr 2005

d Ministério da Saúde. Portaria nº 1.459, de 24 de junho de 2011. Institui no âmbito do Sistema Único de Saúde – SUS – a Rede Cegonha. *Diario Oficial Uniao*; 27 jun 2011
